# Dialysis Membranes Influence Perfluorochemical Concentrations and Liver Function in Patients on Hemodialysis

**DOI:** 10.3390/ijerph15112574

**Published:** 2018-11-17

**Authors:** Wen-Sheng Liu, Hsiang Lin Chan, Yen-Ting Lai, Chih-Ching Lin, Szu-Yuan Li, Chih-Kuang Liu, Han-Hsing Tsou, Tsung-Yun Liu

**Affiliations:** 1 Division of Nephrology, Department of Medicine, Taipei City Hospital, Zhongxing Branch, Taipei 10466, Taiwan; robertliu2001@yahoo.com; 2 School of Medicine, National Yang-Ming University, Taipei 10466, Taiwan; lincc2@vghtpe.gov.tw (C.-C.L.); syli@vghtpe.gov.tw (S.-Y.L.); 3 Institute of Environmental and Occupational Health Sciences, School of Medicine, National Yang-Ming University, Taipei 10466, Taiwan; a12602426@hotmail.com; 4 College of Science and Engineering, Fu Jen Catholic University, New Taipei city 24451, Taiwan; 5 Department of Child Psychiatry, Chang Gung Memorial Hospital and University, Taoyuan 33043, Taiwan; ivyhlin70@kimo.com; 6 Department of Physical Medicine and Rehabilitation, National Taiwan University Hospital Hsin-Chu Branch, Hsinchu 30041, Taiwan; csmclaiyt@gmail.com; 7 Department of Nursing, Yuanpei University, Hsinchu 30041, Taiwan; 8 Division of Nephrology and Department of Medicine, Taipei Veterans General Hospital, Taipei 10466, Taiwan; 9 College of Medicine and Graduate Institute of Business Administration, Fu Jen Catholic University, New Taipei City 24451, Taiwan; DAC24@tpech.gov.tw; 10 Institute of Food Safety and Health Risk Assessment, National Yang-Ming University, Taipei 10466, Taiwan

**Keywords:** perfluorochemicals, hemodialysis, dialysis membrane, polysulfone, LC-MS/MS

## Abstract

*Introduction*: Perfluoro-octanesulfonate (PFOS) and perfluoro-octanoic acid (PFOA) are two toxic perfluorochemicals (PFCs) commonly used as surfactants. PFCs are difficult to be eliminated from the body. We investigated the influence of different dialysis membranes on the concentrations of PFCs in patients under hemodialysis. *Method*: We enrolled 98 patients. Of these, 58 patients used hydrophobic polysulfone (PS) dialysis membranes, and the other 40 had hydrophilic membranes made by poly-methyl methacrylate (PMMA) or cellulose triacetate (CTA). Liquid chromatography tandem mass spectrometry coupled was used with isotope dilution to quantify PFOA and PFOS. *Results*: The predialysis concentrations of PFOA and PFOS in patients with hydrophobic PS dialysis membranes were 0.50 and 15.77 ng/mL, respectively, lower than the concentrations of 0.81 and 22.70 ng/mL, respectively, in those who used hydrophilic membranes (such as CTA or PMMA). Older patients have higher PFOS and poorer body function, with lower Karnofsky Performance Status Scale (KPSS) scores. The demographic data of the two groups were similar. However, patients with hydrophobic PS dialysis membranes had lower predialysis aspartate transaminase (AST) (*p* = 0.036), lower glucose levels (*p* = 0.017), and better body function (nonsignificantly higher KPSS scores, *p* = 0.091) compared with patients who used other membranes. These differences may be associated with the effects of different membranes, because PFOA positively correlated with AST, while PFOS negatively correlated with body function. *Conclusions*: This is the first study comparing PFC levels in uremic patients with different dialysis membrane. PS membrane may provide better clearance of PFCs and may, therefore, be beneficial for patients.

## 1. Introduction

Perfluorochemicals (PFCs) are hydrophobic toxicants and have a high persistence in the environment. Perfluoro-octanesulfonate (PFOS) and perfluoro-octanoic acid (PFOA) are two common surfactants, used in leathers, food packages, and textiles [1]. These two PFCs comprise the majority of all PFCs and can bioaccumulate and biomagnify in humans [2]. These compounds are hard to eliminate due to enterohepatic cycling [3]. Drinking water, wrapping material, and food can be potential exposure routes for PFCs.

These PFCs have shown harmful effects in animals and humans. In animal studies, PFCs are associated with abnormal neural behavior and even cancer [1,4]. Human studies showed intrauterine PFC exposure is associated with low birth weights [5]. Further evidence has shown that exposure to PFCs is related to decreased sperm counts, the equilibrium status of glucose and lipids, abnormal endocrine function, increased carotid artery intima–media thickness in adolescents and lower bone density in women [6,7,8,9].

Due to these toxic effects, Sweden declared PFOS a “persistent organic pollutant” in the Stockholm Convention in 2005. In 2007, the United States Environmental Protection Agency came to agreement with factories on decreases in manufacturing PFOA and discontinuation of all PFOA by 2015 [10]. But many developing countries are still producing these PFCs [11].

PFCs are mainly eliminated from the body by the kidneys. The membrane slit of a glomerulus is about 50 nm, while the diameters of PFOA and PFOS are about 300 nm. PFCs are secreted by organic anion transporters on the renal tubular cells from serum to urine instead of simple filtration at glomerulus [12]. However, 90% of the hydrophobic PFCs bond with serum albumin in the human body and are difficult to excrete in the urine. In humans, PFOA and PFOS have long half-lives of about 3.8 years and 5.4 years, respectively [13]. Toxic substances may be easily accumulated in uremic patients due to renal tubular cells damage. Thus, patients with renal disease are more likely to suffer from accumulation of PFCs and further toxicity.

The main treatment for renal failure is hemodialysis (HD) [14]. There were more than 70,000 (about 0.3% of the total population) patients under dialysis in Taiwan by 2015, and this population keeps increasing [15]. The main causes of mortality in patients with chronic kidney disease (CKD) are cardiovascular diseases (CVD). In previous studies, PFOS was highly correlated with high cholesterol levels and poor performance status, which implies a poor prognosis [16,17].

There are several types of dialysis membranes with different physical qualities. It is not known whether the type of dialysis membrane affects the concentration of PFCs in uremic patients. There are two categories of dialysis membranes, hydrophobic and hydrophilic. Dialysis membranes made by polysulfone (PS) are more hydrophobic than those made of cellulose triacetate (CTA) or poly-methyl methacrylate (PMMA) [18]. Studies showed differences in platelet activation and biocompatibility with different membranes [19]. The membranes are considered to cause differences in endotoxin adsorption and clearance of different particles [20]. Membrane properties might influence PFC concentrations.

We investigated the relation of different dialysis membranes and concentrations of PFCs and clinical profiles in uremic patients. We used liquid chromatography tandem mass spectrometry (LC-MS/MS) with isotope dilution to quantify PFOA and PFOS in the serum of patients under HD, and compared the differences.

## 2. Materials and Methods

### 2.1. Ethics Statement

This study was approved by the Institutional Review Board/Ethics Committee of Taipei Veterans General Hospital (201010007IC) before the trial began. Participants gave informed consent in accordance with the Declaration of Helsinki.

### 2.2. Inclusion and Exclusion Criteria

In this cross-sectional study, we included patients who had received maintenance HD therapy thrice weekly for more than 3 months. Patients who had transplantations or peritoneal dialysis were excluded. Patients who had blood transfusions or intravenous lipid nutrition supplements, chemotherapy, antibiotics, or immunosuppressant were also excluded. A total of 98 patients were included. Of these, 58 used a hydrophobic membrane made of PS, while 40 used a hydrophilic membrane composed of CTA (*N* = 11) or PMMA (*N* = 29).

### 2.3. Data Collection

Patient characteristics and clinical data such as age, gender, time of each dialysis session, cause of renal failure (diabetes), and duration of hemodialysis therapy were obtained directly from the medical records. Hemodialysis treatment profiles (time of each HD session, initial and maintenance doses of anticoagulants blood flow and dialysate flow, artificial kidney surface area, urea reduction ratio, Kt/V, clearance) were obtained from medical records. Laboratory parameters were gathered at the beginning of the month prior to hemodialysis. Physical function of patients were evaluated by a physician using the Karnofsky performance status (ranging from 0 (death) to 100 (fully normal functioning)) [21].

In this study, coupled LC-MS/MS was used with isotope dilution to quantify PFOA and PFOS in the serum. The differences in PFOA and PFOS levels in the blood before and after dialysis were evaluated, and clinical profiles were also analyzed. Blood samples before and after dialysis were collected at a teaching hospital in northern Taiwan. We used LC-MS/MS to quantify PFOA (*m*/*z* 413→369), and PFOS (*m*/*z* 499→80) as the main particles for PFOA and PFOS for further analysis.

### 2.4. Measurement of PFOA and PFOS

All blood samples were stored at –80 °C before analysis. The frozen sera were thawed at 4 °C and vortex-mixed for 30 s to reach homogeneity. A serum sample of 50 µL in a polypropylene centrifuge tube was then vortexed with 50 µL of 1% formic acid (pH 2.4) for 30 s. Afterwards, 1 µL of a 10 µg/mL internal standard solution (^13^C_4_-PFOA and ^13^C_4_-PFOS, Wellington Laboratories Inc., Guelph, ON, Canada) and 40 µL of acetonitrile were added to each sample before further vortexing. These samples were sonicated for 20 min and centrifuged at 18,000× *g* for 20 min. The supernatants were collected and filtered through a 0.22 µm polyether sulfone syringe filter to a screw-cap vial.

In this study, the LC-MS/MS system used comprised of an Agilent 1100 series system (Agilent Tech., Santa Clara, CA, USA), coupled to a Finnigan TSQ Quantum Discovery Max spectrometer system (Thermo Electron Corporation, Breda, The Netherlands). The electron spray ionization source was in negative ion mode. LC-MS/MS and isotope dilution were carried out simultaneously for quantification of PFCs.

A sample of serum (5 µL) was injected onto a 2.0 × 150 mm Capcell Pak^®^ 3 µm C18 column (Shiseido Co., Tokyo, Japan). The mobile phases consisted of 10 mM ammonium acetate in water (A) and pure acetonitrile (B) delivered at a constant flow rate of 0.2 mL/min. After injection, the mobile phase was kept at 30% B for 3 min. Then the gradient of B was increased to 65% in 3 min. Afterwards, the gradient of B was gradually increased in 5 min to 100% B, where it was kept for 7 min. The column was set at 30% B for 1.5 min.

Optimized mass spectrometry parameters were as follows: spray ion voltage 3000 V, capillary temperature 210 °C, sheath gas pressure 10 arbitrary units, auxiliary gas pressure 5 arbitrary units, ion sweep gas pressure 4 arbitrary units, collision gas pressure 1.0 mTorr, and dwell time 100 ms. Detection was carried out in selective reaction monitoring (SRM) mode. The monitored collision energy (V) and SRM transitions were as follows: 10 V, *m*/*z* 413→369 for PFOA; 12 V, *m*/*z* 417→372 for ^13^C_4_-PFOA; 40 V, *m*/*z* 499→80 for PFOS; 40 V, *m*/*z* 503→80 for ^13^C_4_-PFOS.

### 2.5. Statistical Analysis

The sample size of uremic patients was determined based on an effect size to detect the significance of differences in concentrations of PFCs in different membrane groups. If we permitted a 5% chance of a type I error (α = 0.05), with a power of 80%, assuming the difference of PFCs among two different dialysis-membrane groups was at least half of the standard derivation, then approximately 64 uremic patients would be required for a sufficient sample size. Sera from 98 uremic patients were collected in this study.

Statistical analysis was done using SPSS statistical software (version 19.0; IBM, Armonk, NJ, USA). Distributions of continuous variables in groups were expressed as mean ± SD and compared by Student’s *t*-test. A *p* value less than <0.05 was considered statistically significant.

We analyzed the differences in predialysis concentrations of PFOA and PFOS in patients with different dialysis membranes by independent *t*-test, as well as the postdialysis concentrations of PFCs. If the variable is not normally distributed, we used the Mann–Whitney *U* test to compare the difference between different groups. The concentrations of PFOA and PFOS in uremic patients before and after HD were analyzed by paired *t*-test (Table 1).

The chi-square (χ^2^) test was used for categorical variables such as gender, comorbidities (diabetes mellitus), and medical treatments (such as hypertension treatment or iron-supplement use).

The correlations of PFOA and PFOS in uremic serum with patient characteristics, hemodialysis treatment, hemogram, and biochemical profile were analyzed by linear regression.

## 3. Results

The mean concentrations of PFOA and PFOS in all uremic patients were 0.62 (SD = 0.27) and 18.60 (SD = 48.92) ng/mL, respectively, before dialysis. After dialysis, the concentration of PFOA was 0.54 ng/mL (SD = 0.29) with no significant change, but PFOS was significantly reduced to 1.97 ng/mL (SD = 0.91).

**Figure 1:**
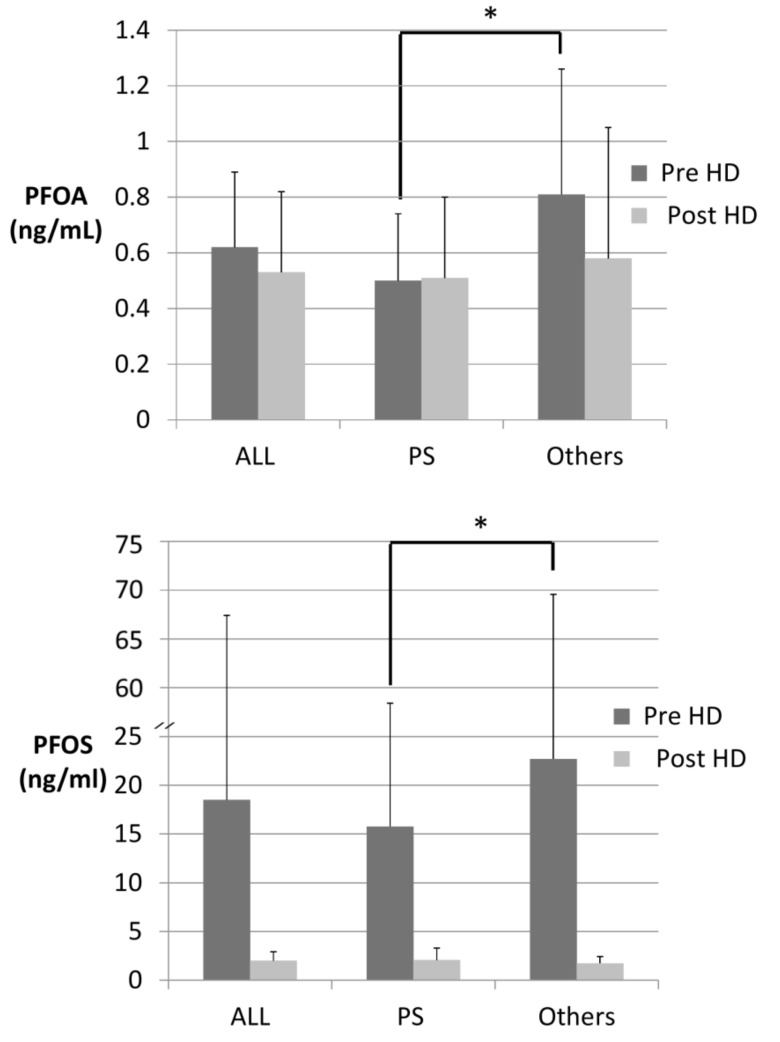
Concentrations of PFOA and PFOS in all patients, patients with PS membranes and patients with other hydrophilic membranes before and after dialysis. Patients with hydrophobic PS membranes had lower PFOA (*p* = 0.001) and PFOS (*p* = 0.026) levels than patients with other dialysis membranes before dialysis (* *p* < 0.05).

The levels of PFOA and PFOS before dialysis in patients with hydrophobic PS membranes were 0.50 (SD = 0.24) and 15.77 (SD = 42.64) ng/mL; in the other patients with hydrophilic membranes, these levels were 0.81 (SD = 0.45) and 22.70 (SD = 46.86) ng/mL (Table 1). PFC levels were much lower in patients with hydrophobic PS membranes than other membranes (*p* < 0.001 for PFOA and *p* = 0.026 for PFOS). Postdialysis PFC levels showed no significant differences in patients with different membranes (Figure 1).

**Figure 2:**
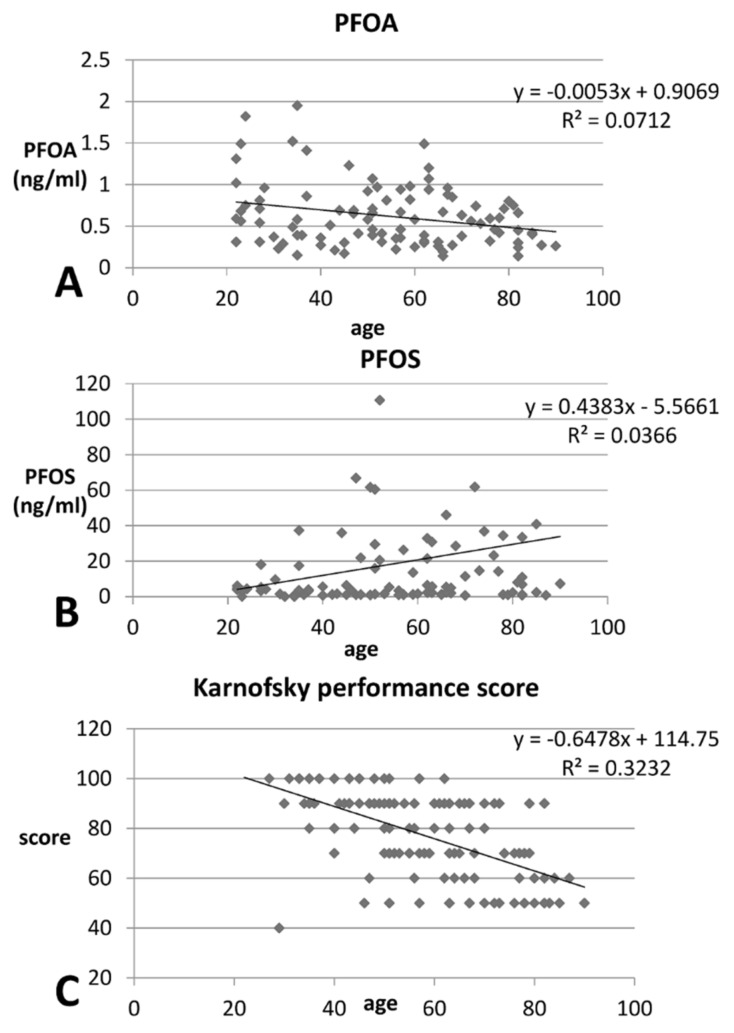
Linear regression of age with the concentration of (**A**) predialysis PFOA (*p* = 0.083), (**B**) PFOS (*p* = 0.008), and (**C**) Karnofsky performance status (*p* < 0.001) in all uremic patients.

In linear regression, age was postiviely correlated with PFOS (*p* = 0.008), but negatively correlated with Karnofsky performance status scores (*p* < 0.001). (Table 2) (Figure 2) This indicates that higher concentrations of PFOS are more likely to be found in older uremic patients, and body function significantly declines with age.

Among our patients, 56.1% were men, and the average age was 55.85 years (SD = 14.79). (Table 3 and Table 4) No significant differences were noted in the two groups with different membranes, including dialysis duration, except fewer patients were on iron supplements (43.1% vs. 57.5%, *p* = 0.016) (Table 3). Compared with the hydrophilic membrane group (Table 4), the body-function score (Karnofsky performance status) was higher (although not significantly) in patients with hydrophobic PS membranes (77.60 vs. 71.82, *p* = 0.091). Further analysis before and after dialysis showed no significant differences in clinical profiles between groups with different membranes except for lower AST (21.61 vs. 26.0 U/L, *p* = 0.036) and lower glucose (124.73 vs. 149.71 mg/dL, *p* = 0.017) levels before dialysis in the PS group (Table 5).

**Figure 3:**
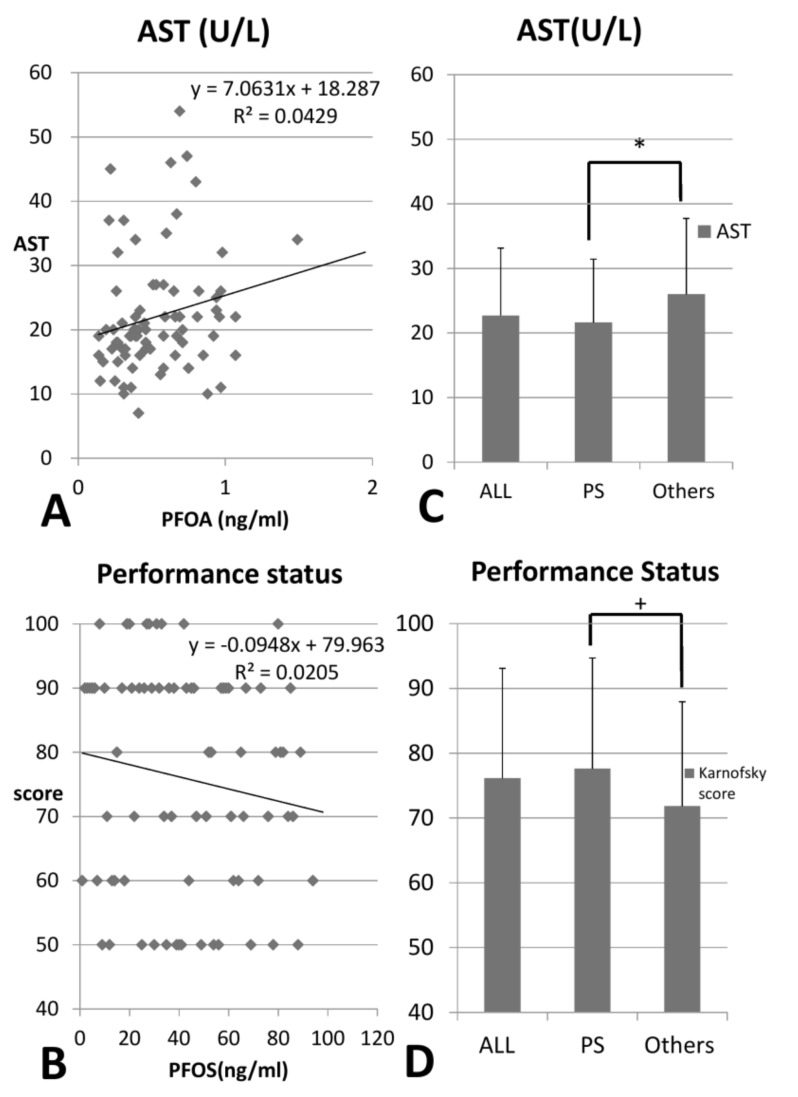
(**A**) PFOA positively correlated with AST (*p* = 0.047) and (**B**) PFOS negatively correlated with body function (*p* = 0.008) by linear regression. Patients with other hydrophilic membranes had (**C**) higher AST (*p* = 0.036) and (**D**) nonsignificantly lower Karnofsky performance status (*p* = 0.091) than patients with PS dialysis membranes. (* *p* < 0.05, ^+^ *p* < 0.10).

Patients with hydrophilic membranes, compared with patients with hydrophobic PS membranes, had higher predialysis AST and nonsignificant lower performance status score (*p* < 0.05) (Figure 3).

## 4. Discussion

PFC (PFOA and PFOS) concentrations were much lower in the serum of patients with hydrophobic PS membranes than patients with hydrophilic membranes (CTA and PMMA) before dialysis (PFOA: 0.50 vs. 0.81 ng/mL, *p* < 0.001; PFOS: 15.77 vs. 22.70 ng/mL, *p* = 0.026). This may because PFOA and PFOS are both hydrophobic materials and are more easily cleared using hydrophobic membranes. Previous studies showed membrane properties have a considerable influence on inflammation, platelet activation, and phagocytes [22]. To the best of our knowledge, this is the first study that proves the hydrophobic or hydrophilic properties of membranes can infuence the concentrations of environemental pollutants.

We found age was posivitely correalated with PFOS (*p* = 0.008), but negatively correlated with PFOA (*p* = 0.083, although not significantly) and Karnofsky performance status (*p* < 0.001) (Figure 2). This is reasonable, in that body function declines with age, and this decline is more rapid in patients with CKD [23]. Uremic patients are more prone to inflammation and easily suffer from malnutrition. [17] PFOS has been manufactured for longer than PFOA, and its concentration in patients’ serum was much higher than that of PFOA. Older patients have had longer exposure to PFOS than younger patients; hence, PFOS levels are likely to be higher in older people [24].

Age does not have significant positive correlation with PFOA according to our study (Table 2). One possible explanation is that PFOA has not yet been banned in developing countries like Taiwan and China [25]. In modern society, younger generations may encounter many more products with PFOA than in previous years. Studies showed that PFC levels are higher in children than in adults in Taiwan [26]. In 2014, PFC levels in serum samples from Taiwanese children was also higher than that observed between 2006 and 2008 [27]. However, more studies are needed to investigate the relation between PFC value and the age of affected people. In Taiwan, the serum concentration of PFOA was 3.22 ng/mL and that of PFOS was 8.52 ng/mL in healthy people, which are similar to levels in other countries (PFOA: 1.5 ± 10 ng/mL, PFOS: 5.0 ± 35 ng/mL) [26].

Further analysis showed that PFC correlated with age only in patients with hydrophilic membranes (age was negatively, but not significantly, correlated with PFOA, *p* = 0.082) and positively correlated with PFOS, *p* = 0.049) (Table 2). There was no correlation of PFCs with age in patients with hydrophobic PS membranes, possibly due to better clearance.

There was no differences in patient characteristics and treatment profiles between groups, except for patients with hydrophobic PS membranes were less likely to be on iron supplements than their counterparts (43.1% vs. 57.5%, *p* = 0.016). No difference was found between the PS group and hydrophilic group for gender, comorbidity, average dialysis duration or dialysis cleanance, erythropoiesis stimulation agent dosage, or medication for hypertension [28] (Table 3 and Table 4).

In a previous study in 2018 [15], PFOS was significantly correlated with ferritin in patients under HD. PFOS may bind with iron-store protein and interfere with iron transport or storage. Patients with other hydrophilic memebranes may have a disturbance of iron utilization due to higher PFC concentrations in their body compared with those who use PS membranes, and hence, be more likely to be on iron supplements. However, more studies are needed to prove this hypothesis (Table 1).

All complete blood counts and biochemical profiles before HD showed no significant differences between these two groups except for lower AST (AST: 21.61 vs. 26.0 U/L, *p* = 0.036) and glucose levels before dialysis (124.73 vs. 149.71 mg/dL, *p* = 0.017) in patients with hydrophobic membranes. These biochemical differences may be explained by the lower PFOA in uremic patitents using the PS mambrane. A previous study in 2009 showed that PFOA is associated with metabolic syndrome and hyperglycemia in adolescents and adults. [7] This may also imply that PFOA poses a harmful effect on the liver due to liver control in glucose homeostasis [29]. An animal study in 2015 also proved PFOA may induce liver lesion [30] (Figure 3).

Patients with hydrophobic PS membranes had no significant age difference compared with the other group (59.49 vs. 61.83, *p* = 0.283). However, their performance status were better, although not significantly (77.60 vs. 71.82, *p* = 0.091) (Table 4). This may result from better compatibilty of the hydrophobic PS dialysis membrane with the properties yof PFCs, resulting in higher clearance with a low accumulation of PFCs. Hence, these patients had better physical function. Higher Karnofsky performance status also implies better survival in hemodialysis patients [31]. In a previous study, PFOS was postively correlated with cholesterol level, and negatively correlated with Karnofsky performance status [15,32,33]. This indicates that PFOS is correlated with CVD with a poor prognosis among uremic patients because the Karnosfsky performance status score is a powerful predictor of patient survival [34,35].

In summary, we found that concentrations of PFOA and PFOS are influenced by dialysis-membrane properties. Uremic patients with hydrophobic PS dialysis membranes had lower PFC levels (PFOA, *p* < 0.001, and PFOS, *p* = 0.026), were less likely be on Fe supplements (*p* = 0.016), but had nonsignificant higher Karnofsky performance status scale (*p* = 0.091), and lower AST (*p* = 0.036) and glucose levels before dialysis (*p* = 0.017) than patients who used other kinds of membranes. We hope our study can raise awareness of environmental toxins in uremic patients [25]. PFC clearance may differ with different dialysis membranes in uremic patients.

The limitations of our study included the small number of patients, and the fact that no significant difference could be obtained if patients were divided into more groups for comparison. More studies with a larger number of patients may provide better insight into how different dialysis membranes affect PFCs or other persistent organic pollutants in uremic patients as, the CKD population keeps increasing in the world, accounting for 11%–13% of total population [15]. This would potentially contribute to an important and still unexplored issue for public health.

## 5. Conclusions

This is the first study that investigated the relation of PFCs in uremic patients with different dialysis membranes. It proved cartain membranes provide better clearance of PFCs and may therefore be more beneficial for patients than other membranes. Further study is needed to investigate the correlation.

## Figures and Tables

**Table 1:** PFOA and PFOS concentrations in uremic patients with different membranes before and after dialysis.

	All (*N* = 98)		PS (58)		Others (40)		PS vs. Others
ng/mL	Mean	±SD	Mean	±SD	Mean	±SD	*p*
* PFOA Pre-HD	0.62	0.27	0.50	0.24	0.81	0.45	<0.001
Post-HD	0.54	0.29	0.51	0.29	0.58	0.47	0.372
paired *t*-test (p)	0.707		0.828		0.753		
* PFOS Pre-HD	18.60	48.92	15.77	42.64	22.70	46.86	0.026
^+^ Post-HD	1.97	0.91	2.08	1.22	1.74	0.69	0.055
* paired *t*-test (p)	0.001		0.017		0.017		

* *p* < 0.05, ^+^ *p* < 0.10. Pre-HD: before hemodialysis, post-HD: after hemodialysis, PS: polysulfone, PFOA: perfluoro-octanoic acid, PFOS: perfluoro-octanesulfonate, others: artificial kidney with other kinds of membrane.

**Table 2:** Linear regression of age with PFOA and PFOS in predialysis serum and Karnofsky performance status.

	All (*N* = 98)		AK: PS (*N* = 58)		AK: others (*N* = 40)	
Age	β	*p*	β	*p*	β	*p*
^+^ PFOA (pre-HD)	−14.19	0.083	5.61	0.540	−14.18	0.082
* PFOS (pre-HD)	0.422	0.008	0.360	0.304	0.654	0.049
* Karnofsky performance status	−0.65	<0.001	−0.63	<0.001	−0.58	0.007

* *p* < 0.05, ^+^ *p* < 0.10; AK: artificial kidney membrane.

**Table 3:** Characteristics and treatment profiles of uremic patients under hemodialysis.

All (*N* = 98)		(%)	PS (*N* = 58)	(%)	Others (*N* = 40)	(%)	*p*
Men	55	56.1%	33	56.9%	22	55.0%	1.00
DM	56	57.1%	36	62.1%	20	50.0%	0.233
HTN medication	18	18.4%	14	24.1%	4	10.0%	0.101
Hepatitis	19	19.4%	12	20.7%	7	17.5%	0.807
* Iron supplement	48	48.9%	25	43.1%	23	57.5%	0.016

* *p* < 0.05; DM, diabetes mellitus; HTN: hypertension.

**Table 4:** Patient characteristics and treatment profile with different AK.

All (*N* = 98)	Mean	±SD	PS Mean	(*N* = 58) ± SD	Other Mean	(*N* = 40) ± SD	*p*
Age (year)	55.85	14.79	59.49	14.94	61.83	13.65	0.283
HD duration (month)	59.75	67.75	58.42	72.81	52.10	50.63	0.64
EPO (×10^4^ U) dosage/month	2.09	1.02	2.12	1.06	1.99	0.86	0.526
^+^ Karnofsky performance status	76.12	16.97	77.60	17.09	71.82	16.10	0.091
Blood flow (mL/min)	272.18	33.36	273.30	36.46	268.79	21.47	0.390
Dialysate flow (mL/min)	510.15	56.99	511.50	62.71	506.06	34.82	0.636
HD frequency (/week)	2.98	0.12	2.98	0.14	3.00	0.00	0.417
Dialysis time (hours)	4.023	0.37	4.045	0.39	3.96	0.29	0.221
AK surface size (m^2^)	1.90	0.27	1.91	0.28	1.86	0.23	0.294
AC Initial dose (×10^3^ U)	1.32	0.98	1.36	0.96	1.20	1.05	0.444
AC maintenance dose(×10^3^ U)	0.22	0.27	0.24	0.29	0.16	0.21	0.260
Urea reduction ratio	0.74	0.05	0.75	0.05	0.74	0.05	0.282
Kt/V (Gotch)	1.39	0.22	1.40	0.23	1.34	0.20	0.251
Kt/V ((Daugirdes))	1.64	0.29	1.66	0.30	1.60	0.26	0.374
Ccr (mL/min)	6.03	2.00	6.01	1.99	6.11	2.08	0.797
nPCR	1.02	0.26	1.01	0.24	1.04	0.30	0.477
TAC urea	37.20	10.20	36.84	9.82	38.14	11.23	0.536

^+^*p* < 0.10; AC: anticoagulant, Kt/V: dialysis adequacy, Ccr: creatinine clearance, nPCR: normalized protein catabolic rate, TAC urea: time average concentration for urea, EPO: erythropoietin.

**Table 5:** Hemogram and biochemical profile with different membranes before dialysis.

All (*N* = 98)	Mean	±SD	PS Mean	(*N* = 58) ± SD	Others Mean	(*N* = 40) ± SD	*p*
WBC (×1000/uL)	6.83	2.46	6.71	2.53	7.17	2.25	0.355
RBC (×10^6^/uL)	3.36	0.50	3.37	0.50	3.30	0.50	0.447
Hb (g/dL)	9.89	1.20	9.93	1.14	9.75	1.37	0.460
Hct (%)	30.39	3.67	30.52	3.54	30.01	4.08	0.497
MCV (fL)	91.17	7.23	90.94	7.34	91.87	7.00	0.522
Platelets (×1000/uL)	195.71	68.31	190.95	66.26	210.15	73.35	0.162
Cholesterol (mg/dL)	154.70	35.57	152.87	35.07	160.21	37.02	0.306
Triglycerides (mg/dL)	136.62	78.87	141.10	85.42	123.18	53.57	0.260
* Glucose (mg/dL)	131.26	52.86	124.73	48.08	149.71	61.58	0.017
Total protein (gm/dL)	6.94	3.98	6.58	0.78	6.62	0.99	0.833
Albumin (gm/dL)	3.92	0.37	3.94	0.36	3.85	0.40	0.209
Globulin	2.95	4.02	25.7	0.90	2.68	1.33	0.575
* AST (IU/L)	22.70	10.45	21.61	9.82	26.00	11.72	0.036
ALT (IU/L)	18.77	10.88	18.70	11.04	19.00	10.55	0.891
Alk-P (IU/L)	93.72	83.06	93.86	91.75	93.30	49.16	0.974
Total bilirubin (mg/dL)	0.54	0.15	0.55	0.17	0.52	0.10	0.311
Uric acid (mg/dL)	6.99	1.18	7.03	1.23	6.88	1.06	0.506
Sodium (mEq/L)	138.92	2.73	138.70	2.71	139.56	2.71	0.114
Potassium (mEq/L)	4.56	0.66	4.51	0.63	4.72	0.72	0.100
Cl (mEq/L)	98.83	5.62	98.46	5.32	99.91	6.38	0.202
Calcium (mg/dL)	9.27	0.86	9.26	0.86	9.33	0.85	0.702
Phosphorus (mg/dL)	4.69	1.33	4.64	1.30	4.86	1.41	0.406
Creatinine (mg/dL)	9.18	2.22	9.18	2.20	9.16	2.32	0.959
BUN (mg/dL)	61.69	17.09	61.79	16.38	61.41	19.37	0.913
Fe (ug/dL)	59.39	22.15	59.04	22.54	60.43	21.26	0.753
UIBC (ug/dL)	188.99	55.09	187.15	55.07	194.39	55.62	0.510
TIBC (ug/dL)	248.38	48.10	246.19	47.47	254.82	50.09	0.368
Ferritin (ng/mL)	488.95	422.60	443.26	435.96	465.67	386.28	0.791
TSAT (%)	24.78	9.93	24.90	10.41	24.45	8.49	0.822
Al (ng/mL)	15.16	9.85	14.84	8.98	16.57	13.85	0.680
iPTH (pg/mL)	205.26	301.44	208.98	325.22	194.77	225.21	0.817

* *p* < 0.05; WBC: white blood cells, RBC: red blood cells, Hb: hemoglobulin, Hct: hematocrit, MCV: mean corpuscular volume, AST: aspartate aminotransferase, ALT: alanine transaminase, Alk-P: alkaline phosphatase, Cl: chloride, BUN: blood urea nitrogen, UIBC: unbound-iron binding capacity, TIBC: total iron-binding capacity, TSAT: transferrin saturation, A1: aluminium, iPTH: intact parathyroid hormone.
